# Live time-lapse dataset of *in vitro* wound healing experiments

**DOI:** 10.1186/s13742-015-0049-6

**Published:** 2015-02-25

**Authors:** Assaf Zaritsky, Sari Natan, Doron Kaplan, Eshel Ben-Jacob, Ilan Tsarfaty

**Affiliations:** 1 Department of Cell Biology, UT Southwestern Medical Center, Dallas, TX 75024 USA; 2 Department of Clinical Microbiology and Immunology, Sackler School of Medicine, Tel Aviv University, Tel Aviv, 69978 Israel; 3 Israel Institute for Biological Research, P.O.B. 19, Ness Ziona, 74100 Israel; 4 School of Physics and Astronomy, The Raymond and Beverly Sackler Faculty of Exact Sciences, Tel-Aviv University, Tel-Aviv, 69978 Israel; 5 Center for Theoretical Biological Physics, Rice University, Houston, TX 77005-1827 USA; 6 Research & Development Unit Assaf Harofeh Medical Center, Zerifin, 70300 Israel

**Keywords:** Collective cell migration, Wound healing assay, HGF/SF-Met, Live cell imaging, Image analysis

## Abstract

**Background:**

The wound healing assay is the common method to study collective cell migration *in vitro*. Computational analyses of live imaging exploit the rich temporal information and significantly improve understanding of complex phenomena that emerge during this mode of collective motility. Publicly available experimental data can allow application of new analyses to promote new discoveries, and assess algorithms’ capabilities to distinguish between different experimental conditions.

**Findings:**

A freely-available dataset of 31 time-lapse *in vitro* wound healing experiments of two cell lines is presented. It consists of six different experimental conditions with 4–6 replicates each, gathered to study the effects of a growth factor on collective cell migration. The raw data is available at ‘The Cell: an Image Library’ repository. This Data Note provides detailed description of the data, intermediately processed data, scripts and experimental validations that have not been reported before and are currently available at GigaDB. This is the first publicly available repository of live collective cell migration data that includes independent replicates for each set of conditions.

**Conclusions:**

This dataset has the potential for extensive reuse. Some aspects in the data remain unexplored and can be exploited extensively to reveal new insight. The dataset could also be used to assess the performance of available and new quantification methods by demonstrating phenotypic discriminatory capabilities between the different experimental conditions. It may allow faster and more elaborated, reproducible and effective analyses, which will likely lead to new biological and biophysical discoveries.

**Electronic supplementary material:**

The online version of this article (doi:10.1186/s13742-015-0049-6) contains supplementary material, which is available to authorized users.

## Data description

The wound healing assay, the traditional method to study collective cell motility and migration in medicine and biology, is performed by following the closure of a wound formed by scratching a confluent cell culture. The scratch is imaged and measured periodically during the healing process, and the rate of change in the wound’s area is recorded and compared with those in other cells and treatments. Quantifying the change in wound area has been well established for over 20 years, but recent advances demonstrated the ability to use live time-lapse data to capture phenotypes that are invisible to the naked eye (e.g., refs [1-6]) and may have future implications in screening [1,3,7].

To our knowledge, the only public repository available for collective cell migration setting was produced by Simpson et al. [8], who tested how wound healing dynamics of MCF-10A breast epithelial cells alter in response to targeting by small interfering RNAs (siRNAs). This time-lapse data is severely limited in their statistics. First, a single time lapse for one gene knockdown does not allow significant statistical confidence (i.e., two conditions cannot be distinguished with N = 1). Second, the imaged field of view contains only a small (<200) number of cells limiting statistical sufficiency to quantify the collective behavior. These limitations are addressed here by presenting a dataset that includes 4–6 independent experiments for each condition, each with a large field of view with at least 1,000 cells.

Here we present a public dataset of live wound healing time-lapse data. The experiments were designed to study the spatiotemporal dynamics of *in vitro* wound healing, particularly the effects of Hepatocyte Growth Factor/Scatter Factor (HGF/SF) [9,10], as a model system to determine the effects of a growth factor on collective cell migration [2,11]. Two cell lines were examined: Madin-Darby Canine Kidney epithelial cells (MDCK), the line commonly used for *in vitro* collective migration studies, and the mouse mammary adenocarcinoma line D1-DMBA-3 (DA3). DA3 cells were either left untreated or treated with HGF/SF ([9,12]) or the Met inhibitor PHA665752 [13] (PHA henceforth) with or without HGF/SF. MDCK cells were either left untreated or treated with HGF/SF. All raw image data is publicly available at ‘The Cell: an Image Library’ [14-16] and detailed in Table 1. A total of 21 DA3 experiments (6 Control, 5 + HGF/SF, 4 PHA, 6 PHA + HGF/SF) and 10 MDCK experiments (5 Control, 5 + HGF/SF) were deposited. Intermediate processed data that includes the monolayer contours, the estimated motion-fields and scripts for spatiotemporal analysis are available via the GigaDB database [17] and detailed in Table 2.

**Table 1:** **Summary of the raw data included in this Data Descriptor and their access number at ‘The Cell: an Image Library’ repository** [15,16]

Cell line	Treatment	N	CIL numbers
DA3	Control	6	CIL43406-43411
DA3	+HGF/SF	5	CIL43401-43405
DA3	PHA	4	CIL45451-45454
DA3	PHA + HGF/SF	6	CIL43412-43417
MDCK	Control	5	CIL44501-44502
			CIL44506-44508
MDCK	+HGF/SF	5	CIL44503-44505
			CIL44509-44510

**Table 2:** **Summary of the raw and intermediately processed data included in this Data Descriptor and experiments name in GigaDB**

Cell line	Treatment	N	Experiment names in GigaDB
DA3	Control	6	SN29_L1, SN29_L5, SN29_L6, SN77_L1, SN77_L2, SN77_L8
DA3	+HGF/SF	5	SN29_L8, SN29_L9, SN29_L10, SN29_L11, SN29_L12
DA3	PHA	4	SN77_L20, SN77_L28, SN77_L29, SN77_L30
DA3	PHA + HGF/SF	6	SN77_L33, SN77_L35, SN77_L36, SN77_L38, SN77_L40, SN77_L41
MDCK	Control	5	DKWH7_L1, DKWH7_L3, DKWH7_L10, DKWH7_L15, DKWH7_L16
MDCK	+HGF/SF	5	DKWH7_L4, DKWH7_L5, DKWH7_L6, DKWH7_L18, DKWH7_L19

The experimental methodology to generate this dataset is expanded from previous descriptions [2,11] and is detailed in Additional file 1. It has further been validated, curated and annotated as detailed below. DA3 and MDCK cell lines were selected because they are well characterized for their HGF/SF induced biological activities as well as their Met expression and signaling, the molecular focus of our studies (Additional file 2). HGF/SF concentration and PHA dosage was determined as detailed in Additional file 3, which also includes information regarding blindness, data curation and annotation. Additional file 4 details the intermediate processed data and the scripts available in the GigaDB database [17].

We previously used this data to propose a generalization scheme to quantify *in vitro* collective migration assays using classification for phenotypic discrimination between these different experimental conditions [2]. More recently, we used this data to describe the propagation of strain rate, directionality and coordination to cells located in deeper layer of the cell sheet [11].

The raw data presented here can be reused to (a) evaluate the classification-power of new quantitative measures on different experimental conditions, (b) reproduce our results or (c) find additional insights. Below we suggest several alternative uses.

In ref [2] we used spatiotemporal measures of speed or texture to distinguish between control, +HGF/SF-treated and PHA + HGF/SF-treated DA3 cells via classification. The updated dataset additionally includes DA3 cells treated with PHA and MDCK cells (Control, +HGF/SF). This data can be used to look for differences between more conditions or across the two cell lines. It would also be useful to investigate different measures to characterize changes between cell lines or different conditions (e.g., refs [1,3,18]).

Repetitions of control experiments (six for DA3 cells, five for MDCK cells) allow searching for intrinsic phenotypes that emerge during collective cell migration. The complete dataset allows investigation of the role of HGF/SF as a model growth factor in collective cell migration. We find the following open questions to be important and suggest that the presented dataset be used to study them:

### Late stage of the wound healing process

We have previously focused on the initial stages of wound healing, from the initial scratch until first contact between cells from opposing borders of the wound [11]. Very limited analysis was dedicated to the later stages of the healing process [2], an open arena that can further be investigated using the presented dataset.

### Single cell analysis

Density plays a major role in collective migration: denser cells move slower but more coordinately (e.g., in refs [4,5]. However, in the current dataset cells seem to become sparser and faster [2] but more coordinated [11] as response to HGF/SF. Cell density was estimated based only on a few cells that were manually annotated and no direct single cell analysis was performed because the measures were calculated for a grid of subcellular-sized local patches. New investigations can focus on the dynamics of cell density and their relation to different motility phenotypes for the two cell lines or under HGF/SF-Met signaling.

### Characterizing cells in coordinately migrating clusters

We recently introduced a method to detect spatial clusters of cells that migrate coordinately within the monolayer [11]. How these cells’ dynamics differ from less-coordinated cells remains an open question that may help understand intercellular coordination and can be addressed using the presented dataset.

## Availability and requirements

- Project name: Time-lapse in vitro wound healing experiments
- Project home page: https://github.com/gigascience/paper-zaritsky2015
- Operating systems: Platform independent
- Programming language: Matlab
- Other requirements: Matlab
- License: GNU GPL v3
- Any restrictions to use by non-academics: none

## Availability of supporting data

The datasets supporting the results of this article are available in the *GigaScience* repository, GigaDB [17]. The raw data is also available at ‘The Cell: an Image Library’ repository [15,16].

## Additional files

Additional file 1:
**Supporting Methods.**
Additional file 2:
**Literature-based Validations of Cell Line Selection.**
Additional file 3:
**Technical Validation.**
Additional file 4:
**Intermediately Processed Data available at GigaDB Database.**

## Abbreviations

DA3: Mouse mammary adenocarcinoma line D1-DMBA-3
HGF/SF: Hepatocyte Growth Factor / Scatter Factor
MDCK: Madin-Darby Canine Kidney epithelial cells
