# Supplementary material for: Live time-lapse dataset of in vitro wound healing experiments
Source: Gigascience. 2015 Feb 25;4:8. doi: 10.1186/s13742-015-0049-6 (PMC4341232; doi:10.1186/s13742-015-0049-6)
Supplement: Additional file 1: — Supporting Methods. [file 13742_2015_49_MOESM1_ESM.pdf]

Additional File 1: Supporting Methods

The methods here are expanded from previous descriptions in [1] and [2].

Cell lines and treatments: Two cell lines were used: D1-DMBA-3 (DA3) derived from the mouse mammary adenocarcinoma, and Madin-Darby Canine Kidney (MDCK) epithelial cells. DA3 cells were untreated (denoted Control), treated with 50ng/ml HGF/SF (denoted +HGF/SF), treated with 2.5μM of the Met inhibitor PHA665752 [3] (denoted PHA) or treated with 2.5μM PHA together with 50ng/ml HGF/SF (denoted PHA+HGF). MDCK cells were untreated or treated with 50ng/ml HGF/SF. Wound healing assay [4] was used as a trigger to collective migration. To isolate the various biological effects, cells were starved (0.1% FCS) before and during the assay.

Cultures: DA3 parental cell line and induced in BALB/C mice by dimethylbenzanthracene [5] and DA3 cells expressing the fluorescent protein mCherry [6] were maintained in DMEM supplemented with 10% heat-inactivated fetal calf serum (FCS, Gibco-BRL) in a 37°C, 5% CO_2 incubator. MDCK epithelial cells expressing YFP-membrane were established by stable transfection of YFP-Mem (pEYFP-Mem, Clontech Laboratories Inc, Mountain View, CA, USA) via electroporation using the same method as described in Golan et. al. [7]. These cells were maintained in DMEM supplemented with 5% fetal FCS in a 37°C, 5% CO_2 incubator. Cell proliferation and scatter assays demonstrated no significant difference between the parental and fluorescent labeled MDCK and DA3 cell lines (data not shown).

Wound healing assay: Cells were grown to 90% confluence in 24-well plates (Costar (R) plates, Corning, NY, USA). Prior to scratching, the cells were starved by

changing the medium to DMEM containing 0.1% FCS (starvation medium) for 4 hours (DA3) or 24 hours (MDCK). The medium was then changed to either fresh starvation medium (Control), starvation medium with 50 ng ml^{-1} HGF/SF (+HGF/SF), starvation medium with 2.5 µM ($2.5*10^{-6}$ Molar) PHA for an additional 2 hours (only for DA3 cells, PHA), or starvation medium with HGF/SF and 2.5 µM of PHA for an additional 2 hours (only for DA3 cells, PHA+HGF/SF). A scratch of approximately 300 µm in width was generated using a 200 µl tip [4]. The plate was subjected to time lapse microscopy in a stage incubator (OKOLAB, Italy) on a computer-controlled motorized stage of a confocal microscope (CLSM-510, Carl Zeiss, Germany), used in non-confocal mode, with a 10x (0.30) objective. Image acquisition was initiated 2 hours post scratching. Images were acquired every 14.5 (DA3) or 15.7 (MDCK) minutes for 26 hours (DA3) or 15 hours (MDCK). The coordinates of each scratch were predefined, and a macro that repetitively positions the field of view at each point was executed. The acquired differential interference contrast (DIC) channel of the time-lapse sequence was used for the analysis reported in [1, 2].

Treatment classification: The following generic scheme was applied to quantitatively distinguish between different treatments [1]. Each time-lapse experiment was encoded by a feature-vector that represent a measured physical property (in ref [1] we used spatiotemporal measures of speed and cellular texture). An SVM classifier was trained and tested using "leave one out" validation (due to the small number of experiments obtained) and the output for each experiment was recorded and assessed. This framework can be used to assess the ability of different measures to differentiate between the different treatments.

References

1. Zaritsky A, Natan S, Ben-Jacob E, Tsarfaty I: **Emergence of HGF/SF-Induced Coordinated Cellular Motility.** *Plos One* 2012, **7**.
2. Zaritsky A, Kaplan D, Hecht I, Natan S, Wolf L, Gov NS, Ben-Jacob E, Tsarfaty I: **Propagating waves of directionality and coordination orchestrate collective cell migration.** *PLoS computational biology* 2014, **10:**e1003747.
3. Crosswell HE, Dasgupta A, Alvarado CS, Watt T, Christensen JG, De P, Durden DL, Findley HW: **PHA665752, a small-molecule inhibitor of c-Met, inhibits hepatocyte growth factor-stimulated migration and proliferation of c-Met-positive neuroblastoma cells.** *BMC Cancer* 2009, **9:**411.
4. Liang CC, Park AY, Guan JL: **In vitro scratch assay: a convenient and inexpensive method for analysis of cell migration in vitro.** *Nature Protocols* 2007, **2:**329-333.
5. Fu YX, Watson G, Jimenez JJ, Wang Y, Lopez DM: **Expansion of Immunoregulatory Macrophages by Granulocyte-Macrophage Colony-Stimulating Factor Derived from a Murine Mammary-Tumor.** *Cancer Research* 1990, **50:**227-234.
6. Ofek P, Fischer W, Calderón M, Haag R, Satchi-Fainaro R: **In vivo delivery of small interfering RNA to tumors and their vasculature by novel dendritic nanocarriers.** *The FASEB Journal* 2010, **24:**3122-3134.
7. Golan M, Hizi A, Resau JH, Yaal-Hahoshen N, Reichman H, Keydar I, Tsarfaty I: **Human endogenous retrovirus (HERV-K) reverse transcriptase as a breast cancer prognostic marker.** *Neoplasia* 2008, **10:**521-IN522.
