# Supplementary material for: Live time-lapse dataset of in vitro wound healing experiments
Source: Gigascience. 2015 Feb 25;4:8. doi: 10.1186/s13742-015-0049-6 (PMC4341232; doi:10.1186/s13742-015-0049-6)
Supplement: Additional file 2: — Literature-based Validations of Cell Line Selection. [file 13742_2015_49_MOESM2_ESM.pdf]

Additional File 2: Literature-based Validations of Cell Line Selection

DA3 cells express moderate levels of endogenous Met that rapidly phosphorylates in response to HGF/SF. We previously reported the role that Met plays in these cell lines [1-6]. DA3 cells transfected with Met+HGF/SF are characterized by significantly increased tumorigenic and metastatic activity *in vivo* accompanied by reduced tubule formation. DA3 cells transfected with dominant negative forms of Met (DN-DA3) exhibit reduced Met phosphorylation following exposure to HGF/SF. Furthermore, both *in vitro* scattering and invasiveness and *in vivo* tumorigenicity and spontaneous metastasis levels are reduced [1]. Infection of DA3 cells with Met small-interfering RNA lead to a dramatic reduction of Met expression, also suppresses tumor cell growth and viability *in vitro*, inhibition of HGF/SF-mediated scattering and invasion *in vitro* and led to apoptotic cell death [7].

The role of Met in MDCK cells is widely studied. It was shown that HGF/SF induces cell motility, proliferation, invasiveness, and tubular morphogenesis in these cells [7-25].

It was previously shown that PHA potently inhibits HGF/SF-stimulated and constitutive Met phosphorylation, as well as HGF/SF and Met-driven phenotypes such as cell growth (proliferation and survival), cell motility, invasion, and/or morphology of a variety of tumor cells. In addition, PHA inhibited HGF/SF-stimulated or constitutive phosphorylation of mediators of downstream signal transduction of Met, including Gab-1, extracellular regulated kinase, Akt, signal transducer and activator of transcription 3, phospholipase C γ, and focal adhesion kinase, in multiple tumor cell lines in a pattern correlating to the phenotypic response of a given tumor cell [26].

References

1. Firon M, Shaharabany M, Altstock RT, Horev J, Abramovici A, Resau JH, Vande Woude GF, Tsarfaty I: **Dominant negative Met reduces tumorigenicity-metastasis and increases tubule formation in mammary cells.** *Oncogene* 2000, **19:**2386-2397.
2. Kaplan O, Firon M, Vivi A, Navon G, Tsarfaty I: **HGF/SF activates glycolysis and oxidative phosphorylation in DA3 murine mammary cancer cells.** *Neoplasia* 2000, **2:**365-377.
3. Shaharabany M, Abramovitch R, Kushnir T, Tsarfaty G, Ravid-Megido M, Horev J, Ron R, Itzchak Y, Tsarfaty I: **In vivo molecular imaging of met tyrosine kinase growth factor receptor activity in normal organs and breast tumors.** *Cancer Res* 2001, **61:**4873-4878.
4. Yerushalmi GM, Leibowitz-Amit R, Shaharabany M, Tsarfaty I: **Met-HGF/SF signal transduction induces mimp, a novel mitochondrial carrier homologue, which leads to mitochondrial depolarization.** *Neoplasia* 2002, **4:**510-522.
5. Leibowitz-Amit R, Tsarfaty G, Abargil Y, Yerushalmi GM, Horev J, Tsarfaty I: **Mimp, a mitochondrial carrier homologue, inhibits Met-HGF/SF-induced scattering and tumorigenicity by altering Met-HGF/SF signaling pathways.** *Cancer Res* 2006, **66:**8687-8697.
6. Tsarfaty G, Stein GY, Moshitch-Moshkovitz S, Kaufman DW, Cao B, Resau JH, Vande Woude GF, Tsarfaty I: **HGF/SF increases tumor blood volume: a novel tool for the in vivo functional molecular imaging of Met.** *Neoplasia* 2006, **8:**344-352.
7. Shinomiya N, Gao CF, Xie Q, Gustafson M, Waters DJ, Zhang YW, Vande Woude GF: **RNA interference reveals that ligand-independent met activity is required for tumor cell signaling and survival.** *Cancer Res* 2004, **64:**7962-7970.
8. CANTLEY LG, BARROS EJ, GANDHI M, RAUCHMAN M, NIGAM SK: **Regulation of mitogenesis, motogenesis, and tubulogenesis by hepatocyte growth factor in renal collecting duct cells.** *kidney* 1994, **16:**17.
9. Fukuta K, Matsumoto K, Nakamura T: **Multiple biological responses are induced by glycosylation-deficient hepatocyte growth factor.** *Biochem J* 2005, **388:**555-562.
10. Ishibashi K, Sasaki S, Sakamoto H, Hoshino Y, Nakamura T, Marumo F: **Expressions of receptor gene for hepatocyte growth factor in kidney after unilateral nephrectomy and renal injury.** *Biochemical and biophysical research communications* 1992, **187:**1454-1459.
11. Jia Z, Vadnais J, Lu ML, Noël J, Nabi IR: **Rho/ROCK-dependent pseudopodial protrusion and cellular blebbing are regulated by p38 MAPK in tumour cells exhibiting autocrine c-Met activation.** *Biology of the Cell* 2006, **98:**337-351.
12. Kamei T, Matozaki T, Sakisaka T, Kodama A, Yokoyama S, Peng Y-F, Nakano K, Takaishi K, Takai Y: **Coendocytosis of cadherin and c-Met coupled to disruption of cell-cell adhesion in MDCK cells--regulation by Rho, Rac and Rab small G proteins.** *Oncogene* 1999, **18:**6776-6784.
13. Koch A, Mancini A, El Bounkari O, Tamura T: **The SH2-domian-containing inositol 5-phosphatase (SHIP)-2 binds to c-Met directly via tyrosine residue 1356 and involves hepatocyte growth factor (HGF)-induced lamellipodium formation, cell scattering and cell spreading.** *Oncogene* 2005, **24:**3436-3447.
14. Miao H, Nickel CH, Cantley LG, Bruggeman LA, Bennardo LN, Wang B: **EphA kinase activation regulates HGF-induced epithelial branching morphogenesis.** *The Journal of cell biology* 2003, **162:**1281-1292.
15. Miyake M, Saze K-i, Yaguchi T, Wang J, Suzuta Y, Haga Y, Takahashi SY, Yamamoto Y, Iwabuchi S: **Canine hepatocyte growth factor: molecular cloning and characterization of the recombinant protein.** *Veterinary immunology and immunopathology* 2003, **95:**135-143.
16. Okigaki M, Komada M, Uehara Y, Miyazawa K, Kitamura N: **Functional characterization of human hepatocyte growth factor mutants obtained by deletion of structural domains.** *Biochemistry* 1992, **31:**9555-9561.

17. Santos OF, Barros EJ, Yang X-M, Matsumoto K, Nakamura T, Park M, Nigam SK: **Involvement of hepatocyte growth factor in kidney development.** *Developmental biology* 1994, **163:**525-529.
18. Rosen EM, Nigam SK, Goldberg ID: **Scatter factor and the c-met receptor: a paradigm for mesenchymal/epithelial interaction.** *The Journal of cell biology* 1994, **127:**1783-1787.
19. Santos OF, Nigam SK: **HGF-induced tubulogenesis and branching of epithelial cells is modulated by extracellular matrix and TGF-β.** *Developmental biology* 1993, **160:**293-302.
20. Santos OF, Moura LA, Rosen EM, Nigam SK: **Modulation of HGF-induced tubulogenesis and branching by multiple phosphorylation mechanisms.** *Developmental biology* 1993, **159:**535-548.
21. Tajima H, Matsumoto K, Nakamura T: **Regulation of cell growth and motility by hepatocyte growth factor and receptor expression in various cell species.** *Experimental cell research* 1992, **202:**423-431.
22. Weidner KM, Sachs M, Birchmeier W: **The Met receptor tyrosine kinase transduces motility, proliferation, and morphogenic signals of scatter factor/hepatocyte growth factor in epithelial cells.** *The Journal of cell biology* 1993, **121:**145-154.
23. Hartmann G, Naldini L, Weidner KM, Sachs M, Vigna E, Comoglio PM, Birchmeier W: **A functional domain in the heavy chain of scatter factor/hepatocyte growth factor binds the c-Met receptor and induces cell dissociation but not mitogenesis.** *Proceedings of the National Academy of Sciences* 1992, **89:**11574-11578.
24. Loerke D, le Duc Q, Blonk I, Kerstens A, Spanjaard E, Machacek M, Danuser G, de Rooij J: **Quantitative imaging of epithelial cell scattering identifies specific inhibitors of cell motility and cell-cell dissociation.** *Sci Signal* 2012, **5:**rs5.
25. Poujade M, Grasland-Mongrain E, Hertzog A, Jouanneau J, Chavrier P, Ladoux B, Buguin A, Silberzan P: **Collective migration of an epithelial monolayer in response to a model wound.** *Proc Natl Acad Sci U S A* 2007, **104:**15988-15993.
26. Christensen JG, Schreck R, Burrows J, Kuruganti P, Chan E, Le P, Chen J, Wang X, Ruslim L, Blake R: **A selective small molecule inhibitor of c-Met kinase inhibits c-Met-dependent phenotypes in vitro and exhibits cytoreductive antitumor activity in vivo.** *Cancer research* 2003, **63:**7345-7355.
