# Supplementary material for: Live time-lapse dataset of in vitro wound healing experiments
Source: Gigascience. 2015 Feb 25;4:8. doi: 10.1186/s13742-015-0049-6 (PMC4341232; doi:10.1186/s13742-015-0049-6)
Supplement: Additional file 3: — Technical Validation. [file 13742_2015_49_MOESM3_ESM.pdf]

Additional File 3: Technical Validation

HGF/SF and PHA dosages selection: The concentration of the HGF/SF stock solution was determined by visual assessment of dose depended scatter assay using MDCK cells. As previously described [1], cells were incubated overnight with different dosages of HGF/SF, examined and photographed. 80 ng HGF/SF per ml^{-1} of medium was selected in accordance to the concentration used in the literature [2-4]. HGF/SF isolation and characterization was performed as previously described [5].

PHA inhibition of HGF/SF-Met signaling was reported in the literature (Additional File 2). PHA dosage was determined as follows. First, literature survey suggested a dosage of 5 µM. Then, *in vitro* wound healing experiments were performed to demonstrate that even half this dosage (2.5 µM) is sufficient to effectively reduce wound healing rate (Fig. S1).

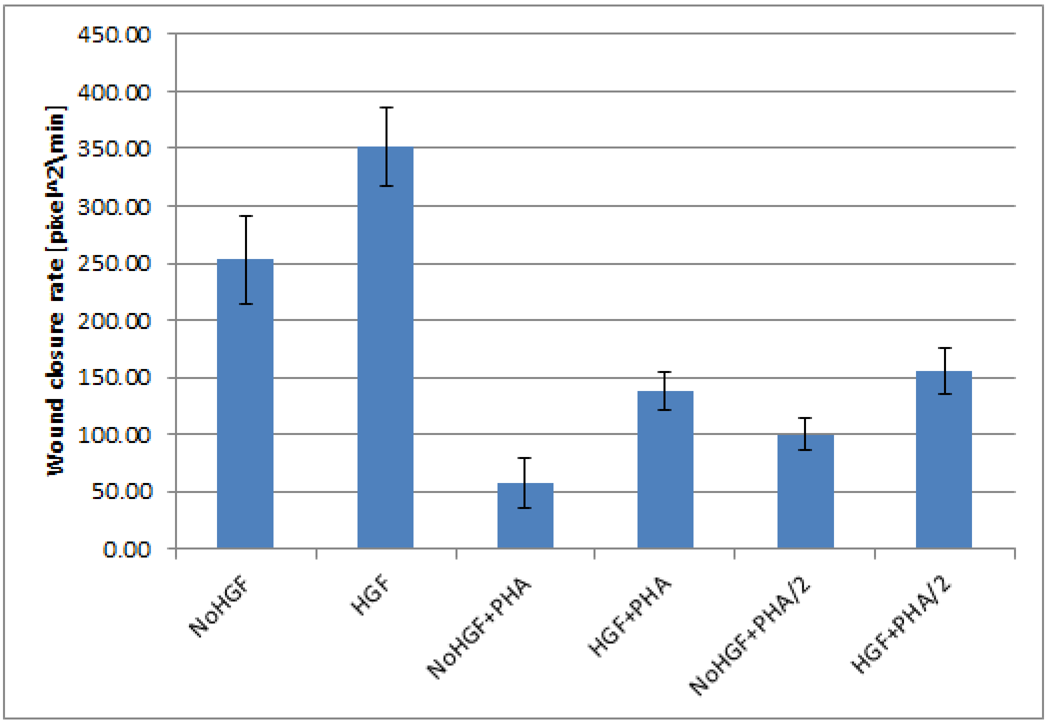

Figure S1. PHA dosage selection. Wound healing rate demonstrate that for HGF/SF treated cells, 2.5 µM of PHA is sufficient to significantly reduce healing rate (pval < 0.0006) to the

level of cells treated with 5 μM of PHA (pval < 0.5). No significant statistical difference was found between cells treated with 2.5 and 5 μM of PHA without HGF/SF (pval < 0.14). Each condition was imaged in 3 wells with 4-8 locations in each well. 2-tailed t-test was used for statistical assessments.

Blindness, data curation and annotation: The experimental conditions were blinded from the first author, who performed the analysis and reported the results. Each time lapse experiment was visually inspected prior to processing, and experiments with very poor imaging quality were discarded from the analysis pipeline. Visual validation of image segmentation results excluded experiments that were not segmented properly to cellular and background regions, as this is a critical step in the analysis that significantly affects quantification. All raw data was deposited at "The Cell: an Image Library", annotated and verified by the authors and the repository representative.

References

1. Zaritsky A, Natan S, Horev J, Hecht I, Wolf L, Ben-Jacob E, Tsarfaty I: **Cell Motility Dynamics: A Novel Segmentation Algorithm to Quantify Multi-Cellular Bright Field Microscopy Images.** *Plos One* 2011, **6**.
2. Firon M, Shaharabany M, Altstock RT, Horev J, Abramovici A, Resau JH, Vande Woude GF, Tsarfaty I: **Dominant negative Met reduces tumorigenicity-metastasis and increases tubule formation in mammary cells.** *Oncogene* 2000, **19:**2386-2397.
3. Laser-Azogui A, Diamant-Levi T, Israeli S, Roytman Y, Tsarfaty I: **Met-induced membrane blebbing leads to amoeboid cell motility and invasion.** *Oncogene* 2014, **33:**1788-1798.
4. Natan S, Tsarfaty G, Horev J, Haklai R, Kloog Y, Tsarfaty I: **Interplay between HGF/SF–Met-Ras signaling, tumor metabolism and blood flow as a potential target for breast cancer therapy.** *clinical trials* 2014, **3:**10.
5. Rong S, Oskarsson M, Faletto D, Tsarfaty I, Resau J, Nakamura T, Rosen E, Hopkins 3rd R, Vande WG: **Tumorigenesis induced by coexpression of human hepatocyte growth factor and the human met protooncogene leads to high levels of expression of the ligand and receptor.** *Cell growth & differentiation: the molecular biology journal of the American Association for Cancer Research* 1993, **4:**563-569.
