# Supplementary material for: Live time-lapse dataset of in vitro wound healing experiments
Source: Gigascience. 2015 Feb 25;4:8. doi: 10.1186/s13742-015-0049-6 (PMC4341232; doi:10.1186/s13742-015-0049-6)
Supplement: Additional file 4: — Intermediately Processed Data available at GigaDB Database. [file 13742_2015_49_MOESM4_ESM.pdf]

Additional File 4: Intermediately Processed Data available at GigaDB Database

Methods: The common basic steps in analysis of *in vitro* time lapse data of wound healing experiments include: (1) segmentation of the monolayer contour, and (2) local correlation-based motion estimation.

Several tools and algorithms exist for segmenting the monolayer from the non-cellular regions in an image [1-3], and Particle Image Velocimetry (PIV) methods are usually applied to estimate cells motion [4, 5]. We used MultiCellSeg [6] and local cross-correlation [7, 8] for the data presented herein, but any other method can be easily plugged in instead.

Data: The main directory contain multi-page .tif and .lsm files, each containing the raw differential interference contrast (DIC) images of a single wound healing experiment. Each such experiment has a corresponding directory that holds the data needed for analysis:

- *images* directory holds the raw DIC images separated to single .tif files.
- *MF* directory holds the local velocity data, represented by two matrices (named dxs, dys) that contain the velocity component calculated for x- and y-coordinates correspondingly for each image pixel. The velocity is given in µm per hour, and was calculated for patches of size 15 µm x 15 µm.
- *ROI* directory holds the segmentation data, namely for each image pixel a labeling as cellular or background pixel.

- *experimentParams.mat* is a file holding experimental parameters such as pixel size in microns and time per frame.

A script named speedKymograph.m creates a kymograph per experiment that displays the spatiotemporal dynamics of cell speed as function of time and distance from the monolayer edge. The script accepts as input the main directory name, goes over the .tif/.lsm data (each file holds raw data of a single time-lapse experiment) and uses the intermediately processed data in the corresponding directories to generate the kymograph. One copy of the kymograph is placed in the experimental directory and the other in a directory named speedKymographs at the main directory. Two files are generated in each of the two directories: a .mat file with the kymograph data and a .bmp image for visualization. Please refer to the documentation in the script for further information.

A file named metadata.mat contains a cell-array that links each time-lapse file to an experimental condition (the field 'experimentNames'), physical pixel size (in µm, the field 'pixelSize'), time per frame (the field 'timePerFrame') and frame number when first contact occurred between cells from opposing borders of the wound (what is referred to as "phase 1" in [7], the field 'timePhase1').

References

1. Geback T, Schulz MM, Koumoutsakos P, Detmar M: **TScratch: a novel and simple software tool for automated analysis of monolayer wound healing assays.** *Biotechniques* 2009, **46:**265-274.
2. Deforet M, Parrini MC, Petitjean L, Biondini M, Buguin A, Camonis J, Silberzan P: **Automated velocity mapping of migrating cell populations (AVeMap).** *Nature Methods* 2012, **9:**1081-1083.
3. Zaritsky A, Wolf L, Ben-Jacob E, Tsarfaty I: **Benchmark for Multi-Cellular Segmentation of Bright Field Microscopy Images.** *Submitted* 2013.
4. Poujade M, Grasland-Mongrain E, Hertzog A, Jouanneau J, Chavrier P, Ladoux B, Buguin A, Silberzan P: **Collective migration of an epithelial monolayer in response to a model wound.** *Proc Natl Acad Sci U S A* 2007, **104:**15988-15993.

5. Petitjean L, Reffay M, Grasland-Mongrain E, Poujade M, Ladoux B, Buguin A, Silberzan P: **Velocity Fields in a Collectively Migrating Epithelium.** *Biophysical journal* 2010, **98:**1790-1800.
6. Zaritsky A, Natan S, Horev J, Hecht I, Wolf L, Ben-Jacob E, Tsarfaty I: **Cell Motility Dynamics: A Novel Segmentation Algorithm to Quantify Multi-Cellular Bright Field Microscopy Images.** *Plos One* 2011, **6**.
7. Zaritsky A, Natan S, Ben-Jacob E, Tsarfaty I: **Emergence of HGF/SF-Induced Coordinated Cellular Motility.** *Plos One* 2012, **7**.
8. Zaritsky A, Kaplan D, Hecht I, Natan S, Wolf L, Gov NS, Ben-Jacob E, Tsarfaty I: **Propagating waves of directionality and coordination orchestrate collective cell migration.** *PLoS computational biology* 2014, **10:**e1003747.
